# Blunt Facial Trauma Causing Isolated Optic Nerve Hematoma

**DOI:** 10.1155/2013/235209

**Published:** 2013-01-21

**Authors:** R. Parab, C. I. Fung, Gerrit Van Der Merwe

**Affiliations:** Department of Radiology and Diagnostic Imaging, University of Alberta, and Royal Alexandra Hospital, Edmonton, AB, Canada T5H 3V9

## Abstract

Traumatic optic neuropathy is an uncommon, yet serious, result of facial trauma. The authors present a novel case of a 59-year-old gentleman who presented with an isolated blunt traumatic left optic nerve hematoma causing vision loss. There were no other injuries or fractures to report. This case highlights the importance of early recognition of this rare injury and reviews the current literature and management of traumatic optic neuropathy.

## 1. Introduction

A recent prospective Canadian epidemiological study showed that 0.4% of all trauma patients had traumatic optic neuropathy [1]. Out of the patients who suffered from a traumatic optic neuropathy, two thirds had significant associated head injuries [1]. Isolated traumatic optic nerve hematoma without evidence of any other traumatic injuries or fractures is a very unusual presentation of traumatic optic neuropathy.

Traumatic optic neuropathy is the result of direct or indirect forces causing injury to the optic nerve. Direct trauma refers to trauma precisely to the optic nerve causing transection, avulsion, orbital hemorrhage, orbital sheath hemorrhage, or orbital emphysema [2, 3] whereas indirect injury is caused by the convergence of traumatic forces focused elsewhere, secondarily involving the optic nerve [3]. The injury to the optic nerve may be primary, referring to ischemia caused by shearing and contusion necrosis of the optic nerve at the time of injury [2], or secondary, reflecting posttraumatic injury of the optic nerve due to a self-propagating compartment syndrome caused by edema or hemorrhage in the confined space of the intracanalicular optic nerve sheath [2, 3]. The final common pathway is that optic nerve axonal injury results in retinal ganglion cell death [2, 4] which peaks within two to three weeks [2, 5] and results in permanent vision loss. 

## 2. Case Report

A 59-year-old male presented to a tertiary care center in Edmonton, AB, Canada, with monocular left-sided vision loss and left retrobulbar pain. An hour and a half prior, he had been struck by a piece of heavy equipment at work resulting in a blunt traumatic injury to his left eye. No other symptoms suggestive of head injury were described. Orbital examination revealed left proptosis and swelling. The left pupil was fixed and dilated, measuring 5 mm in diameter. The patient was unable to visualize light with the affected eye, which was also nonreactive. Normal bilateral extraocular muscle movements were maintained.

**Figure 1:**
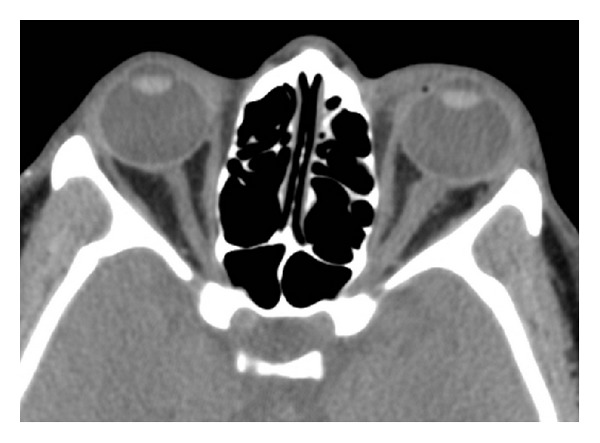
CT orbits demonstrating irregularity to left optic nerve and adjacent fat stranding. Unenhanced CT orbit study demonstrates shaggy irregularity of the distal left optic nerve, which is enlarged compared to the right. Surrounding mild fat stranding is also evident. Minor preseptal soft tissue swelling is apparent with tracking of a thin hematoma along the lateral left orbital wall. The globes are intact, and no additional traumatic injury is visualized.

Unenhanced CT orbits revealed moderate edema and fat stranding surrounding the left optic nerve complex. The distal optic nerve adjacent to the globe measured 7 mm and demonstrated an irregular contour. Additionally, a small preseptal hematoma tracked along the lateral aspect of the globe measuring 4 mm in thickness and extending approximately 1 cm posteriorly along the lateral orbital wall. The left globe was intact. The right optic nerve was normal in caliber, measuring 4 mm in diameter. No other injuries were seen (Figure 1).

Within the clinical context, partial transections of the optic nerve complex or a nerve sheath hematoma were the likely diagnoses. Given the possible diagnosis of a nerve sheath hematoma, the patient was emergently taken to the operating room for nerve sheath fenestration in order to relieve the pressure on the optic nerve. The surgery began approximately 2 hours following presentation to the emergency department and confirmed the presence of a nerve sheath hematoma, which was successfully evacuated. 

## 3. Discussion

In this case, the patient had an indirect injury causing an intraorbital optic nerve hematoma. A primary injury to the optic nerve resulted in nerve shearing and contusion at the time of injury. Secondary insult due to intracanalicular hemorrhage and subsequent compartment syndrome also occurred in this case. These two mechanisms of injury resulted in hemorrhage, ischemia, and pressure necrosis of optic nerve axons and death of retinal ganglion cells, ultimately leading to complete vision loss.

Clinical assessment of these patients focuses on visual acuity, visual field testing, color assessment [2], extraocular muscle movement, pupillary reactivity, direct and indirect ophthalmoscopy, and slit lamp examination [6]. Clinical findings depend on the severity of the injury; in this case, vision loss was caused by indirect trauma to the optic nerve and the resultant death of retinal ganglion cells. The fixed pupil was suspected to be secondary to optic nerve injury and damage to the pupillary constricting short ciliary nerves of the oculomotor nerve. 

CT scans of the orbits are commonly used for assessing patients with traumatic optic neuropathy. The CT scan reveals pathology of the optic nerve and the connective tissue surrounding it which is imperative for surgical planning [2]. MRI can also be used to assess the orbits; however, due to time constrictions faced in emergent situations, MRI is suboptimal for critically ill patients.

Treatment for traumatic optic neuropathy is divided into four groups: conservative management, steroids, decompressive surgery, and combined decompressive surgery and steroids [7]. The rationale behind surgery and steroids is to relieve the compartment syndrome and inflammation, respectively, which cause secondary injury to the optic nerve. Studies comparing treatment options are inconclusive, and due to the relative rarity of the injury, no evidence-based guidelines have been written [2]. The general treatment outlined by Wu et al. [7] limits optic canal decompression to conscious patients, advises methylprednisone dosing of 250 mg every 6 hours for 24–48 hours, and withholds steroids if more than 8 hours from the initial insult.

Given the lack of clinical guidelines, the ophthalmologist on call selected to perform emergent decompressive surgery following CT scan report. Although the nerve sheath hematoma was successfully decompressed, unfortunately, likely due to the delay in presentation, the patient's vision did not immediately improve nor return by period of three months, and he maintains permanent monocular blindness. Our paper reviews the different management strategies of traumatic optic neuropathy and reminds both clinicians and surgeons of the importance of rapid diagnosis and management in this uncommon traumatic injury.

## Abbreviations

CT: Computed tomography
MRI: Magnetic resonance imaging.
